# Screening Method for Polyhydroxyalkanoate Synthase Mutants Based on Polyester Degree of Polymerization Using High-Performance Liquid Chromatography

**DOI:** 10.3390/microorganisms9091949

**Published:** 2021-09-14

**Authors:** Manami Ishii-Hyakutake, Tetsuo Sakurai, Takeharu Tsuge

**Affiliations:** Department of Materials Science and Engineering, Tokyo Institute of Technology, 4259 Nagatsuta, Midori-ku, Yokohama 226-8502, Japan; sakurai.t.ai@m.titech.ac.jp

**Keywords:** polyhydroxyalkanoate (PHA), molecular weight, screening, evolutionary engineering

## Abstract

A high-throughput screening method based on the degree of polymerization (DP) of polyhydroxyalkanoate (PHA) was developed using high-performance liquid chromatography (HPLC). In this method, PHA production was achieved using recombinant *Escherichia* *coli* supplemented with benzyl alcohol as a chain terminal compound. The cultured cells containing benzyl alcohol-capped PHA were decomposed by alkaline treatment, and the peaks of the decomposed monomer and benzyl alcohol were detected using HPLC. The DP of PHA could be determined from the peak ratio of the decomposed monomer to terminal benzyl alcohol. The measured DP was validated by other instrumental analyses using purified PHA samples. Using this system, mutants of PHA synthase from *Bacillus* *cereus* YB-4 (PhaRC_YB4_) were screened, and some enzymes capable of producing PHA with higher DP than the wild-type enzyme were obtained. The PHA yields of two of these enzymes were equivalent to the yield of the wild-type enzyme. Therefore, this screening method is suitable for the selection of beneficial mutants that can produce high molecular weight PHAs.

## 1. Introduction

Plastic pollution in the ocean has emerged as a global concern because it has a significant impact on the marine environment and ecosystems [1]. In particular, microplastics, defined as plastic particles smaller than 5 mm in diameter by the National Oceanic and Atmospheric Administration (NOAA), pose a serious problem because they can easily spread over a wide area and exert adverse effects [2]. Marine biodegradable plastic has, therefore, attracted attention as a solution to this problem because it can be degraded by bacteria in marine environments to CO_2_ and H_2_O [3]. 

Polyhydroxyalkanoate (PHA), a biodegradable plastic, is produced by many kinds of bacteria in nature as an energy storage material from recyclable biomass such as sugars and plant oils [4]. PHA films can be biodegraded in fresh water as well as in seawater [5,6]. As limited types of biodegradable plastics can be degraded in marine environments, PHA is receiving much attention as an alternative to non-biodegradable plastics. Some companies such as Kaneka Corporation in Japan and Danimer Scientific in the US recently launched biosynthesized PHAs in the market [7].

The degree of polymerization (DP) of polymeric materials, including PHA, is one of the factors that determine their physical properties [8]. In general, PHA with high DP (and therefore high molecular weight) is desirable because its molecular weight decreases during downstream processing [9]. A previous study reported that the molecular weight of PHA could be changed by controlling the expression level of PHA synthase, cultivation conditions, and the types of PHA synthase [10,11,12,13]. 

PHA synthases catalyze the polymerization reaction of 3-hydroxyacyl-CoA substrates to PHA polymers. The monomer composition of PHA, which is another factor affecting material characteristics, is often determined by the substrate specificity of the PHA synthase employed [14]. To control the physical properties of PHA, valuable mutants with altered substrate specificity have been generated by evolutionary engineering approaches [15]. Evolutionary engineering experiments mainly comprise two steps as follows: construction of a mutant library and its screening. This approach is beneficial for creating modified enzymes because it can comprehensively and systematically survey the effects of amino acid substitution. However, regarding the DP of PHA, there have been no reports on the evolutionary engineering of PHA synthase to create beneficial enzymes, because a screening method based on the DP of PHA had not been developed. The currently implemented DP determination method involves multiple steps, such as PHA extraction from cells, purification, and analysis using gel permeation chromatography (GPC) or nuclear magnetic resonance (NMR), and the method is generally time-consuming. Therefore, it is not realistic to apply the current DP determination method for evolutionary engineering based on the DP of PHA.

The DP of PHA can be determined from the ratio of the repeating unit to the polymer terminal using high-performance liquid chromatography (HPLC). However, the detection of a polymer terminal poses difficulties in obtaining a clear signal owing to its small abundance relative to the repeating monomer unit. If the signal at the polymer terminal can be clearly detected by HPLC, it may emerge as a high-throughput screening method based on the DP of PHA.

In a previous study, we demonstrated that benzyl alcohol could be introduced at the PHA carboxyl end following addition to the culture medium of recombinant *Escherichia coli* expressing PHA synthase from *Bacillus cereus* YB-4 (PhaRC_YB4_) [16,17]. In this study, we found that benzyl alcohol at the PHA terminal can be detected as a terminal signal by using HPLC. Based on this finding, we developed a novel screening method based on the DP of PHA. Using the screening system, an evolutionary engineering study of PhaRC_YB4_ was conducted, and some PhaRC_YB4_ mutants that produced higher DP of PHA than the wild-type enzyme were acquired.

## 2. Materials and Methods

### 2.1. Materials

Chemicals were purchased from Kanto Chemical Co., Inc. (Tokyo, Japan) and Tokyo Chemical Industry Co., Ltd. (Tokyo, Japan). Poly(3-hydroxybutyrate) (P(3HB)) with an aromatic group at the carboxyl terminal (P(3HB)-93%, number-average molecular weight, *M*_n_ = 9600, benzyl alcohol terminal modification yield = 93%) was biosynthesized as follows: recombinant *E. coli* BW25113 Δ*adhE* (JW1228-KC), an alcohol dehydrogenase deletion strain, harboring pGEM-*phaRC*_YB4_*AB* (carrying *phaRC*_YB4_ from *B*. *cereus* YB-4 and a monomer supplying *phaAB* from *Ralstonia eutropha* H16) [18] was cultured at 37 °C for 72 h in Luria Bertani (LB) medium (10 g/L sodium chloride [NaCl], 10 g/L tryptone, and 5 g/L yeast extract) supplemented with 20 g/L glucose and 1 mL/L benzyl alcohol. PHAs accumulated in bacterial cells were extracted using chloroform and purified with methanol [19]. The molecular weight was determined by GPC, and the terminal modification yield was calculated from ^1^H-NMR spectra [17]. 

### 2.2. Construction of PhaRC_YB4_ Mutant Library

The mutant library of PhaRC_YB4_ from *B*. *cereus* YB-4 was constructed as follows: mutations were introduced by Diversify PCR Random Mutagenesis Kit (TaKaRa Bio Inc., Shiga, Japan) using two primers (forward: 5′-ATGAAAGACCTGCAGGAGAAGGAGATATACATATGACTAC-3′ and reverse: 5′-AAAAAAATAGATCTAACATAGGCTAGTTGG-3′; underlined sequences are *Sse*8387I and *Bgl*II sites, respectively) and pGEM-*phaRC*_YB4_*AB* as a template DNA. As previously described [20], for efficient gene evolution, polymerase chain reaction (PCR) buffer conditions #1, #3, and #5 were selected to introduce 2.0-, 2.7-, and 4.6-bp mutations per 1 kbp, respectively. The PCR product was digested with *Sse*8387I, *Bgl*II, and *Dpn*I and then ligated with *Sse*8387I/*Bgl*II-treated pGEM-*phaRC*_YB4_*AB*.

### 2.3. Screening of Mutants 

**Figure 1:**
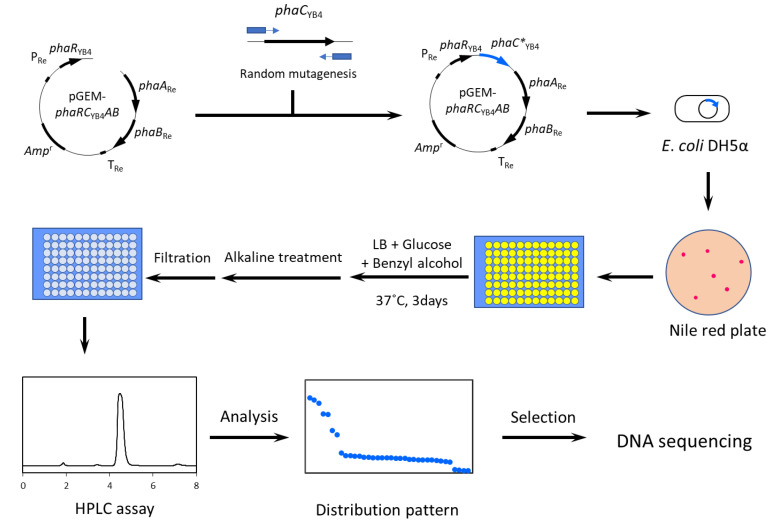
Schematic diagram of the screening method based on the DP of PHA.

Figure 1 shows the schematic diagram of the screening system developed in this study. For the first screening, color selection using Nile red was performed on an agar plate. The ligated solution of pGEM-*phaRC*_YB4_*AB* was used to transform *E. coli* DH5α, and the transformants were plated on Nile red agar containing 100 mg/L ampicillin and 20g/L glucose, incubated at 37 °C overnight, and stored at 4 °C for 1 day [21]. As Nile red is captured in PHA granules in the cell, it can be used as an indicator of intercellular PHA accumulation [22]. That is, PHA-accumulating colonies (here termed “clone”) turn pinkish. Through this step, the clones with inactivated synthase (for example, synthase with a mutation in the active center) could be excluded.

As a secondary screening, 96-well plate cultivation and HPLC analysis were performed. PHA-accumulating transformants, which exhibit orange fluorescence from captured Nile red under 420–500 nm visible light of a Dark Reader transilluminator (MoBiTec, Göttingen, Germany), were inoculated in 0.6 mL LB medium containing 20 g/L glucose, 1 mL/L benzyl alcohol, and 100 mg/L ampicillin in each well of a 96-deep well plate. After cultivation for 3 days at 37 °C, the cells were collected by centrifugation at 1890× *g* (3500 rpm) for 5 min and washed twice with pure water. Then, alkaline treatment was performed by adding 200 μL of 1 N sodium hydroxide (NaOH) to the pellet, followed by heating at 100 °C for 3 h in a dry bath with an aluminum block (TAITEC Co., Saitama, Japan). For more details, see Ref. [21]. After cooling, the reactants were neutralized with 200 μL of 1 N hydrochloric acid (HCl) and centrifuged at 1890× *g* (3500 rpm) for 10 min to precipitate the cell residue. The supernatant was filtered through 96-well filter plates (0.45 μm pore size hydrophilic polytetrafluoroethylene membrane, Pall Co., Port Washington, NY, USA) by centrifugation. The filtrates were collected into new 96-well assay plates and subjected to HPLC analysis. 

HPLC analysis was performed using a Shimadzu LC-10Avp system equipped with an auto-sample injector applicable to 96-well plates. The samples were separated on Fast Acid Analysis Column (100 mm × 7.8 mm I.D., Bio-Rad, Hercules, CA, USA) at 60 °C. As an eluent, 0.014 N sulfuric acid (H_2_SO_4_) containing 20 vol.% acetonitrile (CH_3_CN) was used at a flow rate of 0.7 mL/min. Chromatograms were recorded at a 210 nm wavelength using a UV detector. 

### 2.4. Flask-Scale Cultivation and PHA Analysis

To confirm that the selected clone could synthesize PHA with high DP, flask cultivation was performed. The selected clones were inoculated in 1.7 mL LB medium containing 100 mg/L ampicillin and incubated at 37 °C for 17 h. Next, 1 mL of pre-culture solution was inoculated into 100 mL LB medium containing 20 g/L glucose, 1 mL/L benzyl alcohol, and 100 mg/L ampicillin and incubated at 37 °C for 72 h. After harvesting, the cells were lyophilized. Gas chromatography (GC) was performed to determine the PHA content (wt.%) [23]. PHA accumulated in the cells was extracted using chloroform, and its molecular weight was determined by GPC using two Shodex K-806M joint columns (Showa Denko K. K., Tokyo, Japan). To construct a calibration curve, polystyrene standards with a narrow polydispersity (peak molecular weight *M*_p_ = 1.3 × 10^3^ to 7.3 × 10^6^ g/mol) were used.

### 2.5. Identification of Mutations and Site-Directed Mutagenesis on PhaRC_YB4_

DNA sequencing was performed for the plasmids extracted from the clones selected by screening. DNA sequencing data were analyzed using GENETYX Ver. 11 (GENETYX, Tokyo, Japan). As some mutants possess multiple mutations, enzymes with single amino acid substitutions were generated using site-directed mutagenesis by the overlap extension PCR method [24]. For forward and reverse primers annealing upstream and downstream of the *phaC*_YB4_ gene, *Sse*8387I and *Bgl*II primers described in Section 3.2 were used. Forward and reverse primers containing point mutations near the center of their sequences were also designed (Table S1). Using the corresponding primers, the upstream and downstream sides of the mutations were amplified through a three-step PCR. The resulting fragments served as templates for secondary PCR performed using the *Sse*8387I and *Bgl*II primers to amplify the entire *phaC*_YB4_ region. The resulting *phaC*_YB4_ fragments with point mutations were digested with *Sse*8387I and *Bgl*II and inserted into the same restriction enzyme site of pGEM-*phaRC*_YB4_*AB*. Each construct was transformed into *E. coli* DH5α cells and cultured in flasks under the same conditions as described above.

For clone 13B2, mutation correction was performed on its plasmid pGEM-*phaRC*_YB4_*AB* where *phaC*_YB4_ contains four amino acid substitutions; that is, the constructed plasmid contains three amino acid substitutions.

### 2.6. Evaluation of Acquired Variants

The plasmids pGEM-*phaRC*_YB4_*AB* with point mutations were introduced into *E. coli* JM109. The cells were cultured in LB medium containing 20 g/L glucose and 100 mg/L ampicillin at 37 °C for 72 h. Harvested cells were lyophilized and used for GC analysis as described above for the calculation of PHA content. The molecular weight of PHA was determined by GPC using chloroform-extracted PHAs.

## 3. Results

### 3.1. HPLC Conditions for DP Determination

Del Don et al. [25] reported that P(3HB) was decomposed into crotonic acid, which has strong UV absorption owing to the presence of unsaturated bonds adjacent to the carboxyl group, through alkaline pretreatment using NaOH. As the 3HB unit was quantitatively converted into crotonic acid, the total amount of 3HB could be estimated from the peak intensity of crotonic acid. In contrast, in quantitative terms, the terminal chain units showed an extremely low abundance compared to the repeating unit of 3HB. Therefore, to use the terminal chain unit to estimate the DP of PHA, the terminal structure should provide strong absorption that can be easily detected. As carbon-carbon double bonds, triple bonds, and aromatic groups generally show strong UV absorption, we first measured the spectral absorbance of 3-buten-1-ol with a double bond, 2-propyn-1-ol with a triple bond, and benzyl alcohol with an aromatic group, which could be introduced into the PHA terminal [17]. Benzyl alcohol showed the strongest absorption, which was more than 100 times higher than that of the other compounds (Figure S1). Thus, benzyl alcohol was selected as the terminal structure for detection.

**Figure 2:**
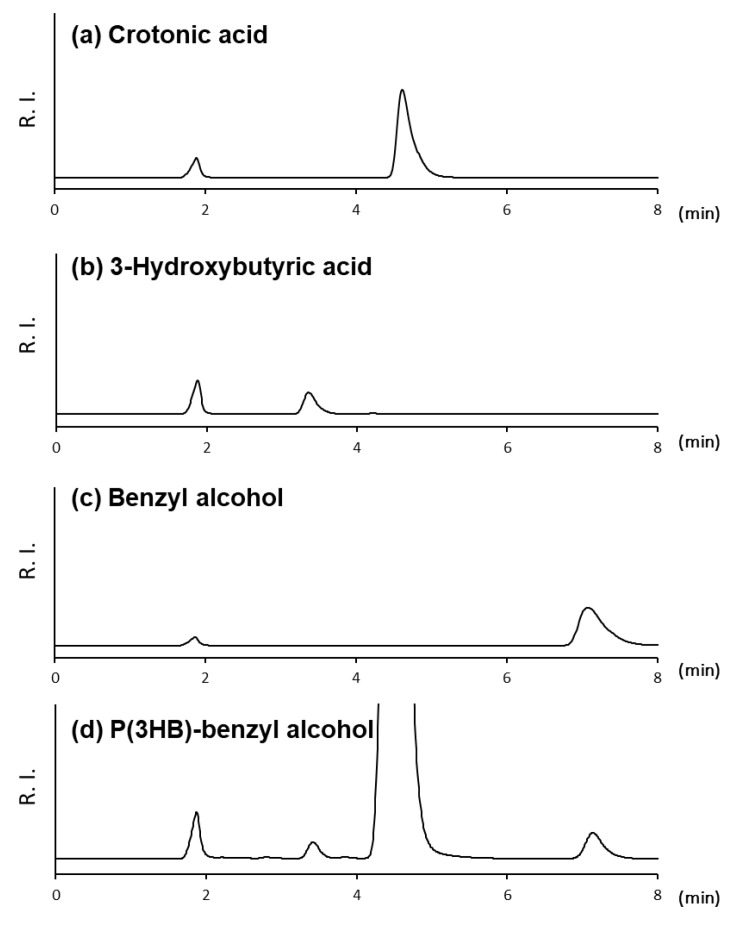
HPLC chromatograms recorded at 210 nm. (**a**) Crotonic acid; (**b**) 3-hydroxybutyrate; (**c**) benzyl alcohol; and (**d**) alkaline-treated P(3HB) with benzyl alcohol end. R. I. means relative intensity.

**Scheme 1:**
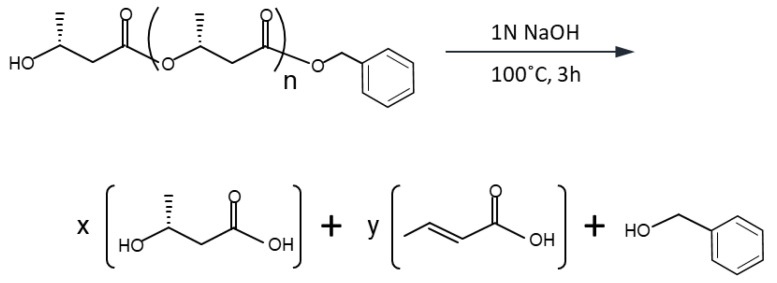
Estimated reaction for alkaline treatment performed in this study. P(3HB) with benzyl alcohol end was converted into 3HB, crotonic acid, and benzyl alcohol. A 3HB repeating unit was converted into both 3HB and crotonic acid (*x*:*y* = 0.64:0.36).

To confirm whether the terminal benzyl alcohol could be detected by HPLC, P(3HB) with benzyl alcohol end was alkaline-treated and analyzed as described in a previous article [21], where the PHA composition was determined by HPLC analysis. The HPLC chromatogram showed one large peak at 4.5 min and two weak peaks at 3.4 and 7.2 min at 210 nm (Figure 2). These retention times corresponded to those of commercially available crotonic acid, 3HB, and benzyl alcohol, respectively. The peak at 1.9 min relates to NaCl produced by the neutralization step. These results indicate that the alkaline treatment of P(3HB) with benzyl alcohol end was proceeded as shown in Scheme 1. The 3HB repeating unit was converted into crotonic acid and 3HB. Hydroxyl- and carboxyl-side terminals were converted into 3HB and benzyl alcohol, respectively. Thus, the detection of the PHA terminal was successful using benzyl alcohol as the terminal structure. 

For rapid analysis, the concentration of acetonitrile in the mobile phase, which affects the retention time of each compound, was considered. Under standard analysis conditions, a mobile phase containing 20 vol.% acetonitrile was used. At a higher acetonitrile concentration (25 vol.%), the peak of crotonic acid overlapped with that of 3HB. Therefore, 20 vol.% acetonitrile was thought to be optimal for this method. Under these conditions, a positive correlation was observed between crotonic acid, benzyl alcohol, and 3HB concentrations and their peak areas (Figure S1). However, the calibration curve slope of 3HB was very small in comparison to the slopes of the other two compounds, indicating that its detection sensitivity was low.

To validate this method, low molecular weight P(3HB) with benzyl alcohol end (93% of terminal modification yield) was analyzed by HPLC and ^1^H-NMR (Table 1). From ^1^H-NMR analysis, the DP of PHA was calculated as the peak intensity ratio of the monomer (3HB repeating unit) to benzyl alcohol. In HPLC analysis, the 3HB repeating unit was converted into both crotonic acid and 3HB, and therefore, the DP of PHA was calculated as the ratio of the total of crotonic acid and 3HB to benzyl alcohol, which was determined from the calibration curves shown in Figure S1. The DPs calculated from ^1^H-NMR and HPLC analyses were 84 and 62, respectively; these similar values prove the applicability of this HPLC method for molecular weight determination. 

The analysis of another P(3HB) with a different DP (DP__NMR_ = 130) revealed that the 3HB repeating unit was converted into crotonic acid at a constant ratio (the yield of crotonic acid was 36%) regardless of its DP. Thus, the DP calculated from the ratio of crotonic acid + 3HB to benzyl alcohol was proportional to that calculated from the ratio of crotonic acid to benzyl alcohol. In addition, the concentration of each compound was proportional to its HPLC peak area. Considering these proportional relationships, DP is easily compared based on the ratio of the HPLC peak area for crotonic acid to that for benzyl alcohol to facilitate the construction of a simplified screening method.

### 3.2. Screening of PhaRC_YB4_ Mutants

**Figure 3:**
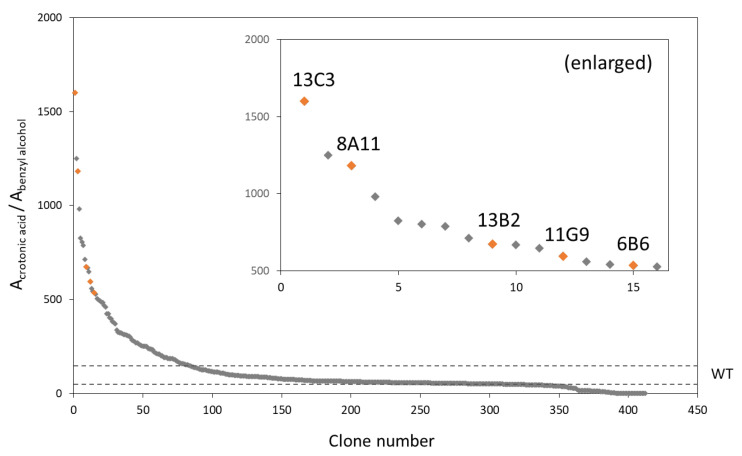
Distribution pattern of HPLC peak ratio in the PhaRC_YB4_ mutant library. Recombinant strains were cultured in 96-well plates and analyzed by HPLC after alkaline treatment. Y-axis is shown as the value of peak area of crotonic acid divided by that of benzyl alcohol. The larger the value, the higher is the DP of PHA. Dashed line represents the range of the result from wild-type.

From 700 colonies grown on Nile red agar plates, 412 clones with relatively strong orange fluorescence were selected and used for 96-well cultivation and HPLC analysis. Figure 3 shows the distribution pattern of the HPLC peak area ratio between crotonic acid derived from the 3HB repeating unit to benzyl alcohol from the PHA terminal. In comparison with the wild-type, the clones that showed remarkably higher values were selected and used in the subsequent flask-scale cultivation step. Most of the selected clones produced higher molecular weight PHA than the wild-type strain. This result indicates that the HPLC-based method constructed in this study could be applied to screen the DP of PHA accumulated in bacterial cells. Five PhaRC_YB4_ mutated clones capable of producing high molecular weight PHA (20- to 300-fold higher than that produced by the wild-type strains) were named 6B6, 8A11, 11G9, 13B2, and 13C3, and were used for further analysis (Table 2). The PHA molecular weight increased up to 2700 × 10^3^. Each clone possessed one to five amino acid substitutions, and some clones possessed additional silent mutations.

### 3.3. Evaluation of Each Point Mutation in Selected Clones

To identify the effect of mutation on the molecular weight of PHA, site-specific mutagenesis was performed on *phaC*_YB4_. Flask-scale PHA production by each mutant is presented in Table 3. The I173N mutant produced a higher molecular weight PHA (*M*_n_ = 270 × 10^3^) than the wild-type (*M*_n_ = 9 × 10^3^), but it was far from that produced by the parent clone 6B6 (*M*_n_ = 2700 × 10^3^, Table 2). This result indicates that both mutations function synergistically. Mutations in clone 13C3 showed a similar trend; that is, the molecular weights of PHA synthesized by single mutation enzymes, P209L and Y328H (*M*_n_ = 250 × 10^3^ and 9 × 10^3^, respectively), were much lower than the molecular weights of PHA synthesized by the parent clone 13C3 (*M*_n_ = 1600 × 10^3^). In fact, the double mutants I173N/E281D and P209L/Y328H produced PHAs with molecular weights similar to those produced by their parent clones. Among the mutations in clone 8A11, only the N207D mutation led to an increase in the molecular weight to 2700 × 10^3^; other single mutations, E211G, N274S, T324A, and K346E had no effect. Clone 11G9 had only one mutation (V94G) and no silent mutation; that is, clone 11G9 was the same as the V94G mutant. This single mutation was clearly related to the molecular weight of PHA.

Each replacement on clone 13B2 was individually corrected to the original amino acid to investigate the substitutional effect (four mutations and six silent mutations). When the 255th amino acid was corrected to Asp, low molecular weight PHA production was observed (*M*_n_ = 47 × 10^3^). The single mutation D255V led to the production of high molecular weight PHA (*M*_n_ = 2700 × 10^3^), which was much higher than that produced by its parent enzyme from clone 13B2 (*M*_n_ = 180 × 10^3^). In the parent enzyme, other mutations might negate the effect of the D255V mutation. 

**Figure 4:**
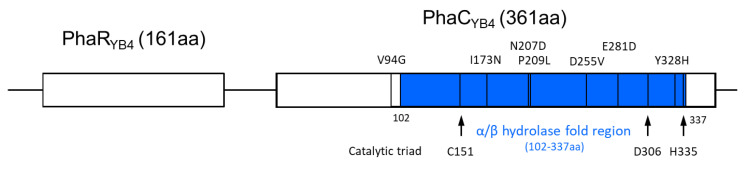
Important mutation on PhaRC_YB4_ to increase the molecular weight. The positions of catalytic triad are shown by arrows. The single mutations of E281D and Y328H did not affect the PHA molecular weight but function synergistically with I173N and P209L, respectively (see Section 3.3).

The effect of each mutation on PHA molecular weight was identified from the relative chain number (Table 3). Here, the chain number of the wild-type enzyme was set to 100. Five mutants, V94G, I173N, N207D, P209V, and D255V, showed a clear reduction in the relative chain number. These and collaborative mutation positions are shown in Figure 4. 

It can be concluded that the amino acid residues V94, I173, N207, P209, and D255 determine the molecular weight of PHA. The amino acid substitutions of V94G, I173N, N207D, P209L, and D255V led to the production of high molecular weight PHAs. In particular, mutants V94G, I173N, and P209L produced PHAs with 28–300-fold higher molecular weights than that produced by the wild-type when grown on glucose and benzyl alcohol, with a yield equivalent to that of the wild-type.

### 3.4. PHA Synthesis by PhaRC_YB4_ Mutant in E. coli JM109

*E. coli* strain JM109 has been used in many studies for the evaluation of different PHA synthases [12,18,27,28]. This strain was used as a PHA production host for further evaluation of the V94G, I173N, and P209L mutants (Table 4). 

All mutants produced higher molecular weight PHA than the wild-type enzyme (26.5- to 42.5-fold higher). In particular, V94G and P209L mutants showed equivalent P(3HB) yields (more than 80% of the wild-type). Therefore, the mutation discovered through the screening method established in this study allows the production of high molecular weight PHAs regardless of the type of *E. coli* host. 

## 4. Discussion

Evolutionary engineering methods have been exploited to generate valuable enzymes with enhanced activity and/or broad substrate specificity [15,20,29]. In previous studies, random mutations were introduced by PCR, treatment with chemical agents such as 3,4-benzopyrene and 1-methyl-3-nitro-1-nitrosoguanidine, or using host strains deficient in primary DNA repair pathways, such as *E. coli* XL1-Red [20,30,31,32,33]. The mutated clones were selected through a screening method based on cell growth, visible color, or rapid analysis using HPLC [21,34,35]. To acquire valuable enzymes by evolutionary engineering methods, a large number of clones should be constructed and tested. For mutant library construction, several established methods and commercialized kits, such as those used in this study (see Materials and Methods), are available. Therefore, the success of evolutionary engineering depends on the construction of an appropriate screening method with a simplified process and increased throughput.

Valuable mutants of PHA synthase have been generated by evolutionary engineering and high-throughput screening. Amara et al. and Hiroe et al. reported a PHA synthase mutant with enhanced productivity that was first screened by color selection using Nile red for hydrophobic PHA [35,36]. Watanabe et al. reported a PHA synthase mutant with broad substrate specificity because the incorporation of a second monomer unit improved the PHA material properties [21]. In the study, PHA monomer composition was rapidly measured using HPLC, and beneficial PHA synthases were selected. In the HPLC analysis, 2-alkenoic acid was generated through the alkaline treatment of 3HA monomers, and its UV absorption derived from unsaturated bonds adjacent to the carboxyl group was detected. 

The DP of PHA is also an important factor for improving PHA material properties [8]. However, evolutionary engineering of PHA synthase has not been conducted using the DP of PHA as an indicator owing to the lack of an appropriate screening method. In general, GPC and NMR are performed to determine the DP of PHA. However, sample preparation for these analyses is time-consuming because PHA should be extracted and purified from bacterial cells.

In this study, we constructed a new screening method based on the DP of PHA by focusing on the detection of the PHA terminal. Even if the terminal was not abundant, its detection was possible by PHA terminal modification using an aromatic compound (benzyl alcohol) that exhibits stronger UV absorption than other alcohols. Unexpectedly, a small peak corresponding to 3HB was detected at approximately 3.4 min (Figure 2) that was thought to be derived from the 3HB repeating unit. Indeed, its concentration was higher than that of crotonic acid (Table 1); however, we decided not to apply this value for screening for the following reasons. First, the 3HB peak was located near the strong peak derived from crotonic acid. Second, the detection sensitivity of 3HB was low because its calibration curve slope was small. Therefore, it was concluded that the 3HB peak is not suitable for quantification.

In this method, the DP of PHA was compared based on the value calculated from the ratio of the HPLC peak area of crotonic acid to the HPLC peak area of benzyl alcohol because the 3HB repeating unit was converted into crotonic acid with constant yield. It should be noted that HPLC analysis provides the DP of PHA with 100% modified carboxyl terminal with benzyl alcohol. Thus, in the case of a low modification yield, it would be far from the actual value. 

To evaluate this screening system, PHA synthase from *B. cereus* YB-4 was selected as the target enzyme because it could introduce benzyl alcohol to the PHA carboxyl terminal with high yield [17]. This synthase showed a high PHA yield, but its PHA molecular weight was low under conditions expressing its alcoholytic activity [18,37,38]. In the case of PhaRC_YB4_, the PHA terminal could be modified with benzyl alcohol via both the chain transfer and alcoholysis reactions. As for other synthases that do not catalyze the alcoholysis reaction, the PHA terminal will be modified with benzyl alcohol because these enzymes also utilize alcohol compounds as chain transfer agents and introduce them into PHA terminals [39,40]. Therefore, the screening method constructed in this study can be applied to other PHA synthases.

Some selected clones that showed high values in the distribution pattern (Figure 3) did not produce much higher molecular weight PHA than that produced by the wild-type enzyme. This result was mainly observed for the weak pinkish colony on the Nile red plate, suggesting that the clone accumulated only a small amount of PHA. The number of benzyl alcohol terminals would decrease along with PHA accumulation, posing a difficulty for detection and consequently leading to inaccurate quantification. The reason why clone 6B6 showed a lower distribution pattern than the other selected mutants (Figure 3), although it could produce the highest molecular weight PHA (*M*_n_ = 2700 × 10^3^), is probably related to its low PHA content (10 wt.%). When constructing a new screening method, it was emphasized to increase the throughput and compromise the quantification accuracy. An accurate analysis such as a flask-scale experiment should be performed after the selection because the selection of wrong colonies could not be avoided in the high-throughput analysis.

Examination of individual point mutations showed that V94, I173, N207, P209, and D255 residues contributed to an increase in the molecular weight of PHA. In general, there is a trade-off between the molecular weight and yield of PHA polymers produced by microbial systems [10,41]. This relationship was observed in the case of PHA production by the D255V mutant. When *E. coli* JM109 is used as a production host, V94G and P209V mutants can overcome this dilemma because they can produce higher molecular weight PHA (more than 26.5-fold as compared to the wild-type) at a production yield equivalent to that of the wild-type (more than 80% of the wild-type enzyme). This molecular weight is similar to that of PhaRC_Bm_ (PHA synthase from *B. megaterium*); however, the PHA yield was much higher than that of PhaRC_Bm_ [18]. In comparison to other synthases that showed similar production yields, these mutants produced relatively higher molecular weight PHAs [12,27,28]. These results highlight the possibility that a slight change in the three-dimensional structure caused by these mutations may enhance the polymerization activity and/or suppress alcoholytic activity. This was achieved because we succeeded in screening numerous mutants using a newly constructed method.

## 5. Conclusions

A novel HPLC screening method for PHA synthase based on the DP of PHA was developed by utilizing the PHA terminal structure. Introduction of aromatic compounds to the PHA terminal led to the successful detection and quantification of the PHA terminal. The new method was applied for the screening of a PhaRC_YB4_ mutant library to select mutants that can polymerize PHA with high DP. Finally, it was revealed that the V94G and P209L mutants produced high molecular weight PHAs with a production yield equivalent to that of the wild-type PhaRC_YB4_.

## Figures and Tables

**Table 1:** Analysis of benzyl alcohol-capped P(3HB) by ^1^H NMR and HPLC.

Polymer	*M*_n_GPC_	^1^H NMR Analysis				HPLC Analysis			
		Relative Ratio		DP__NMR_ ^1^	Modification Yield [%] ^2^	Relative Ratio			DP__HPLC_ ^3^
		Monomer	Benzyl Alcohol			Crotonic Acid	3HB	Benzyl Alcohol	
P(3HB)-93%	9600	84.1	1	84	93	22.56	39.66	1	62

^1^ DP__NMR_ = monomer/benzyl alcohol. ^2^ Terminal modification yield = DP__GPC_/DP__NMR_ × 100. DP__GPC_ = (*M*_n_GPC_) × 0.7/86 [26]. ^3^ DP__HPLC_ = (crotonic acid + 3HB)/benzyl alcohol.

**Table 2:** P(3HB) production by *E. coli* DH5α expressing wild-type or random mutated PhaRC_YB4_.

Synthase	Mutation	Dry Cell wt. (g/L)	P(3HB) Content (wt.%)	Molecular Weight	
				*M*_n_ (×10^3^)	*M*_w_ */M*_n_
WT	-	10.0 ± 0.0	57 ± 5	9 ± 1	1.5
6B6	^173^I(ATC)→N(AAC) ^281^E(GAA)→D(GAT)	2.2 ± 0.2	10 ± 1	2700 ± 210	2.0
8A11 ^1^	^207^N(AAC)→D(GAC) ^211^E(GAA)→G(GGA) ^274^N(AAT)→S(AGT) ^324^T(ACA)→A(GCA) ^346^K(AAA)→E(GAA)	6.5 ± 0.3	41 ± 5	2300 ± 250	2.5
11G9	^94^V(GTG)→G(GGG)	8.0 ± 0.2	53 ± 6	420 ± 24	3.2
13B2 ^1^	^40^Q(CAA)→R(CGA) ^124^F(TTT)→L(CTT) ^255^D(GAT)→V(GTT) ^264^Y(TAC)→F(TTC)	9.9 ± 0.1	62 ± 2	180 ± 28	2.4
13C3 ^1^	^209^P(CCG)→L(CTG) ^328^Y(TAT)→H(CAT)	7.6 ± 0.2	50 ± 1	2400 ± 480	2.6

Cells were cultured in LB medium containing 20 g/L glucose and 1 mL/L benzyl alcohol at 37 °C for 72 h. *M*_n_, number-average molecular weight; *M*_w_, weight-average molecular weight; *M*_w_/*M*_n_, polydispersity index. ^1^ *phaC*_YB4_ gene has silent mutations.

**Table 3:** PHA production of *E. coli* DH5*α* expressing PhaRC_YB4_ with site-directed point mutation.

Mutation Origin	PhaRC_YB4_	Dry Cell wt. (g/L)	P(3HB) Content (wt.%)	Molecular Weight		Relative Chain Number ^1^
				*M*_n_ (× 10^3^)	*M*_w_/*M*_n_	
-	WT	10.0 ± 0.0	57 ± 5	9 ± 1	1.5	100
6B6	I173N	9.2 ± 0.2	51 ± 2	270 ± 32	6.4	2.9
	E281D	8.1 ± 0.1	46 ± 4	7 ± 0	1.7	81
8A11	N207D	7.5 ± 0.1	43 ± 3	2700 ± 260	2.5	0.20
	E211G	8.8 ± 0.1	58 ± 2	11 ± 0	1.7	76
	N274S	8.7 ± 0.2	57 ± 1	13 ± 1	1.8	53
	T324A	10.5 ± 0.0	62 ± 2	9 ± 0	1.7	124
	K346E	10.5 ± 0.2	51 ± 3	8 ± 0	1.6	111
11G9	V94G	8.0 ± 0.2	53 ± 6	420 ± 24	3.2	1.6
13B2	D255V	2.6 ± 0.1	7 ± 1	2700 ± 440	2.2	0.01
13C3	P209L	8.9 ± 0.5	62 ± 2	250 ± 7	4.7	3.6
	Y328H	10.7 ± 0.2	48 ± 9	9 ± 0	1.8	94

Cells were cultured in LB medium containing 20 g/L glucose and 1 mL/L benzyl alcohol at 37 °C for 72 h. *M*_n_, number-average molecular weight; *M*_w_, weight-average molecular weight; *M*_w_/*M*_n_, polydispersity index. ^1^ Relative chain number = (chain number)/(chain number of wild-type). Chain number = (dry cell weight) × (PHA content/100)/(*M*_n_) × *N*_A_. *N*_A_ is Avogadro constant.

**Table 4:** P(3HB) accumulation in *E. coli* JM109 expressing mutated PhaRC_YB4_.

PhaRC_YB4_	Dry Cell wt. (g/L)	P(3HB) Content (wt.%)	P(3HB) Yield (g/L)	Molecular Weight ^1^	
				*M*_n_ (×10^3^)	*M*_w_/*M*_n_
WT ^2^	9.3 ± 0.1	80 ± 1	7.4	20	2.1
V94G	9.5 ± 0.0	63 ± 6	6.0	530	4.9
I173N	9.0 ± 0.1	64 ± 1	5.8	680	5.3
P209L	9.5 ± 0.5	66 ± 2	6.3	850	4.2

Cells were cultured in LB medium containing 20 g/L glucose at 37 °C for 72 h. *M*_n_, number-average molecular weight; *M*_w_, weight-average molecular weight; *M*_w_/*M*_n_, polydispersity index. ^1^ Determined by GPC. ^2^ referred to [18].

## Supplementary Materials

The following are available online at https://www.mdpi.com/article/10.3390/microorganisms9091949/s1, Figure S1: HPLC calibration curves for each compound, Table S1: Primers for site-directed mutagenesis.
