# Supplementary material for: Screening Method for Polyhydroxyalkanoate Synthase Mutants Based on Polyester Degree of Polymerization Using High-Performance Liquid Chromatography
Source: Microorganisms. 2021 Sep 14;9(9):1949. doi: 10.3390/microorganisms9091949 (PMC8469876; doi:10.3390/microorganisms9091949)
Supplement: Supplementary file 1 [file microorganisms-09-01949-s001.zip › Figure S1.pdf]

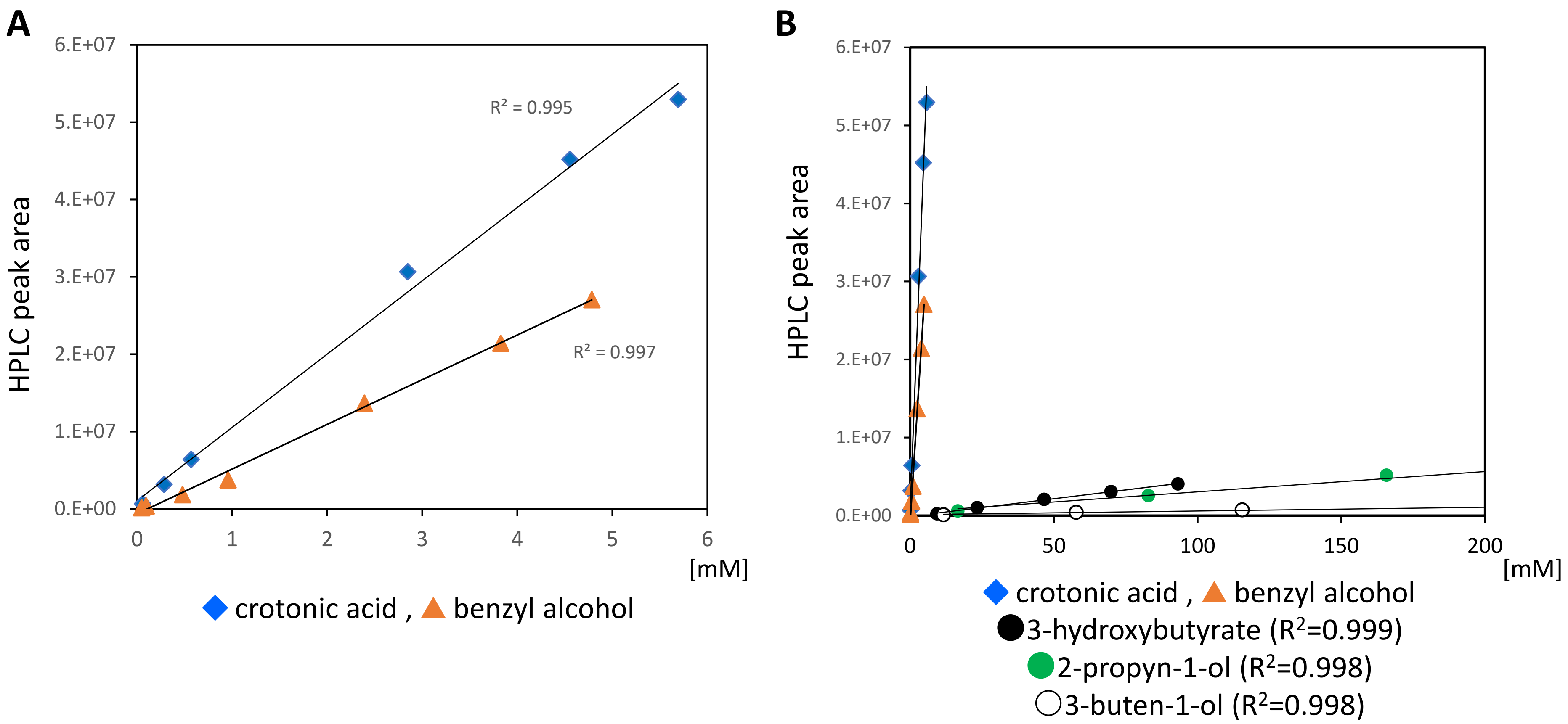

Figure S1. HPLC calibration curves for each compound.
(A) Crotonic acid and benzyl alcohol. (B) Detection sensitivity comparison of each compound.
