# Supplementary material for: Screening Method for Polyhydroxyalkanoate Synthase Mutants Based on Polyester Degree of Polymerization Using High-Performance Liquid Chromatography
Source: Microorganisms. 2021 Sep 14;9(9):1949. doi: 10.3390/microorganisms9091949 (PMC8469876; doi:10.3390/microorganisms9091949)
Supplement: Supplementary file 1 [file microorganisms-09-01949-s001.zip › Table S1.pdf]

Table S1. Primers for site-directed mutagenesis.

Primer Name	Sequence
I173N-F	5′- GCCAATTCGTAACCTAAACTTTATGACAAG-3′
I173N-R	5′- CTTGTCATAAAGTTTAGGTTACGAATTGGC-3′
N207D-F	5′- CGGTTGATACATTTGGAGACATTCCGCCAG -3′
N207D-R	5′- CTGGCGGAATGTCTCCAAATGTATCAACCG-3′
P209L-F	5′- CATTTGGAAACATTCTGCCAGAAATGATTG -3′
P209L-R	5′- CAATCATTTCTGGCAGAATGTTTCCAAATG-3′
E211G-F	5′- CATTCCGCCAGGAATGATTGATTTCGG-3′
E211G-R	5′- CCGAAATCAATCATTCCTGGCGGAATG-3′
D255V-F	5′-GTGGGTTGGTGTTGGTATTCCGTTCCCAGG-3′
D255V-R	5′-CCTGGGAACGGAATACCAACACCAACCCAC-3′
N274S-F	5′-CGTGATTTTTATCAAAGTAATAAATTGG-3′
N274S-R	5′- CCAATTTATTACTTTGATAAAAATCACG-3′
E281D-F	5′- GGTTAAGGGTGATCTTGTGATTCGCGGACA-3′
E281D-R	5′-TGTCCGCGAATCACAAGATCACCCTTAACC-3′
T324A-F	5′-CCATATTTCTAGCGCAGATAAACAATATG-3′
T324A-R	5′- CATATTGTTTATCTGCGCTAGAAATATGG-3′
Y328H-F	5′-CAGATAAACAACATGTATGTTTACCGACAGG-3′
Y328H-R	5′- CCTGTCGGTAAACATACATGTTGTTTATCTG-3′
K346E-F	5′-GGTGGAACAGCTGTAGAACAAACATATCCG-3′
K346E-R	5′- CGGATATGTTTGTTCTACAGCTGTTCCACC-3′
Q40Q-F	5′- CGTGAACCAGAACCACAAGTAGGATTAACG-3′
Q40Q-R	5′-CGTTAATCCTACTTGTGGTTCTGGTTCACG-3′
F124F-F	5′-GAAATTTGATGATTTTGTGTTTGATTATATTGC-3′
F124F-R	5′- GCAATATAATCAAACACAAAATCATCAAATTTC-3′
D255D-F	5′-GTGGGTTGGTGATGGTATTCCGTTCCCAGG -3′
D255D-R	5′-CCTGGGAACGGAATACCATCACCAACCCAC-3′
Y264Y-F	5′-CCAGGTGAATCATACAGACAGTGGATTCG-3′
Y264Y-R	5′- CGAATCCACTGTCTGTATGATTCACCTGG-3′
